# Spatial distribution of benthic macrofauna in the Central Arctic Ocean

**DOI:** 10.1371/journal.pone.0200121

**Published:** 2018-10-17

**Authors:** Andrey Vedenin, Manuela Gusky, Andrey Gebruk, Antonina Kremenetskaia, Elena Rybakova, Antje Boetius

**Affiliations:** 1 P.P. Shirshov Institute of Oceanology, Russian Academy of Sciences, Moscow, Russia; 2 Alfred Wegener Institute, Helmholtz Center for Polar and Marine Research, Bremerhaven, Germany; 3 Max Planck Institute for Marine Microbiology, Bremen, Germany; University of Tasmania, AUSTRALIA

## Abstract

Permanent sea-ice cover and low primary productivity in the mostly ice-covered Central Arctic ocean basins result in significantly lower biomass and density of macrobenthos in the abyssal plains compared to the continental slopes. However, little is known on bathymetric and regional effects on the macrobenthos diversity. This study synthesizes new and available macrobenthos data to provide a baseline for future studies of the effects of Arctic change on macrofauna community composition in the Arctic basins. Samples collected during three expeditions (in 1993, 2012 and 2015) at 37 stations on the slope of the Barents and Laptev Seas and in the abyssal of the Nansen and Amundsen Basins in the depth range from 38 m to 4381 m were used for a quantitative analysis of species composition, abundance and biomass. Benthic communities clustered in five depth ranges across the slope and basin. A parabolic pattern of species diversity change with depth was found, with the diversity maximum for macrofauna at the shelf edge at depths of 100–300 m. This deviates from the typical species richness peak at mid-slope depths of 1500–3000 m in temperate oceans. Due to the limited availability of standardized benthos data, it remains difficult to assess if and how the significant sea-ice loss observed in the past decade has affected benthic community composition. The polychaete *Ymerana pteropoda* and the bryozoan *Nolella* sp. were found for the first time in the deep Nansen and Amundsen Basins.

## Introduction

The deepest parts of the Central Arctic, including the abyssal plains of the Nansen and Amundsen Basins separated by the Lomonossov Ridge, are among the least studied areas of the global oceans. Most surveys of Arctic benthos have been conducted on the shelf or upper slope, whereas data from the deep basins remain rare and scattered in time and space. First data on the deep-sea fauna of the Central Arctic were obtained in 1935 using trawls and dredges not suitable for collecting quantitative samples. Qualitative descriptions of the Central Arctic bathyal and abyssal fauna were made by Soviet expeditions on the vessels *Sadko* in 1935–1937 and *F*. *Litke* in 1955 [1,2]. First quantitative data using mini-LUBS corer and “Okean” grabs were collected in the 1970s in the Canadian Basin from the American Fletcher’s Ice Island [3] and the Soviet drifting station *North Pole-22* [4]. The authors reported extremely low abundances at depths >1000 m of a few individuals per m^2^ and hence a very low benthos biomass of around 0.04 g/m^2^. A recent review of the Arctic macrobenthos confirms the strong decline in biomass by over an order of magnitude from the outer shelves to the inner basins of the Arctic Ocean [5]. The Arctic deep-sea benthos has likely evolved from shallow-water relatives inhabiting the large shelves, with cold temperatures close to freezing point prevailing across the entire depth range. To this date, the communities of the deep basins and the outer shelf share more than half of their taxa [5], and food limitation seems to be the main factor structuring community composition [6].

Today, as the rapid warming and sea ice decline are likely to change the Arctic ecosystem in its entity, comparative analyses of macrofauna community composition, diversity and abundance in space and time remain challenged by the paucity of quantitative, standardized macrofauna data. Sampling surveys were made during expeditions of the research icebreakers *Ymer*, *Polarstern*, *Oden* and *Polar Sea* in the 1990s and 2000s, but unfortunately not according to internationally standardized procedures [7–12]. Over 25 macrobenthic samples were retrieved in 1991 during the *Polarstern* expedition to the Nansen and Amundsen Basins [13,14], but the low volume of samples obtained caused problems in diversity detection [15]. During the *Polarstern* expeditions in 1993 and 1995, nine bathymetric transects were conducted at the northern slopes of Barents, Kara and Laptev Seas from shelf to abyssal depths. Results of these surveys were partly published by [11], [15] and [16]. The present study provides additional analyses of legacy samples from these expeditions and adds a substantial number of new data from surveys during the sea-ice minimum in 2012 [6,17].

Patterns of bathymetric distribution of benthic fauna were previously reviewed for different temperate and tropical ocean regions [18–23]. According to generally recognised patterns, the density and the biomass of macrobenthos decrease with increasing depth due to the declining flux of particulate matter as the main food supply. In contrast, species diversity shows a parabolic pattern with a maximum at depths of 1500–3000 m [24–27]. In the Arctic, the known species diversity of around 1100 taxa does not appear to follow this pattern, and no mid-depth peak of diversity has yet been detected, potentially due to the strongly limited food supply (summarised in [28]).

This hypothesis is supported by recent studies of horizontal distribution patterns of macrofauna in the deep-sea Central Arctic. From the shelf to the deep-sea basins, food supply is declining not only because of increasing water depth, but also because northwards, the sun angle and the sea-ice conditions limit light availability for primary producers. Concentrations of benthic chlorophyll together with macrofaunal biomass are known to be inversely proportional to ice cover [29]. A summary of the abundance, biomass and modelled productivity of the Central Arctic macrofauna was recently published by [6], covering samples from a 20-year period and a depth range of 520–5420 m. It was shown that standing stock and production of the benthic communities are several times higher under the seasonal compared to the multiyear ice zones. This correlation is commonly explained by the higher particle flux under the seasonal ice[6,17].

In the present study we aimed at further testing the effect of location, water depth, sea-ice cover and phytodetritus flux for a large standardized data set of macrofauna at high taxonomical resolution, focusing on the Eurasian slope and basin. [28] noted before that analytic differences among investigators make difficult or even impossible to synthesize species lists from different studies. In our study the entire set of samples was identified by the same specialists. Another aim was to test the entire data set available for any indication of decadal change in community composition between the 1990s and today, i.e. before and after the significant sea-ice decline in this region [30]. We examined the bathymetric distribution patterns of macrofauna along the Barents and Laptev Sea slopes and the spatial distribution pattern within central parts of the Amundsen and Nansen Basins. Our hypotheses were the following: 1) Variations in macrobenthos distribution are controlled by food availability; 2) Community similarity is high across all depth zones; 3) Sea-ice retreat leads to change in the community structure.

## Materials and methods

### Sampling

Material for this study was obtained from three expeditions. During the RV *Polarstern* expedition ARK-IX/4 (August-September 1993), 44 benthic stations were sampled on northern slopes of the Barents and Laptev Seas and adjacent shelf areas and the Nansen Basin. Sampling gears included the multibox-corer (MG) [31] and the 0.25 m^2^ giant box-corer (GKG). From one to fourteen subsamples of the size 0.023 m^2^ were taken from each MG and/or from one to four subsamples of the size 0.022 m^2^ from each GKG using the rectangular frame. Part of these samples was processed previously at the Zoological Museum in St. Petersburg, Russia, and results were published by [15]. The material from additional 27 stations sampled in 1993 was kept untouched until recently. This material has now been analyzed and is used in the present study to obtain a more comprehensive set of baseline data. Information on these stations, including the number of subsamples and sampling area, are shown in Table 1; the entire dataset can be downloaded from doi.pangaea.de/10.1594/PANGAEA.890152. The uppermost 1 cm of sediment from each subsample was washed through a 0.2 mm sieve; the deeper 5 cm were washed through a 0.5 mm sieve. The washed subsamples were preserved in 4% formaldehyde solution. For additional information see [9].

**Table 1:** Station data showing the expedition, geographical position, depth, number of subsamples and sampling area at each station.

Expedition	Region	Station number	Subsamples	Sampling area (m^2^)	Latitude (decimals)	Longitude (decimals)	Depth (m)
ARK IX/4	Barents	14	5 MG; GKG	0.138	81.705	30.330	2915
	Sea	17	GKG	0.023	82.487	37.740	2566
		19	3 MG	0.069	82.763	40.243	2993
		20	1 MG	0.023	82.400	40.893	2027
		24	2 MG	0.046	82.160	42.045	1021
		25	5 MG	0.115	82.123	42.538	534
		27	5 MG	0.115	82.023	43.577	286
	Laptev	31	GKG	0.023	76.508	133.355	38
	Sea	32	5 MG	0.115	78.710	132.292	2962
		35	13 MG	0.299	78.392	133.160	2151
		38	6 MG	0.138	78.167	133.395	981
		39	6 MG	0.138	78.098	133.513	517
		40	3 MG	0.069	78.072	133.608	191
		41	GKG	0.046	77.910	133.555	73
		43	GKG	0.092	77.405	133.557	54
		44	GKG	0.046	77.030	126.413	92
		47	4 MG; GKG	0.280	77.183	126.223	1026
		48	5 MG	0.115	77.135	126.353	552
		49	5 MG	0.115	77.083	126.223	284
		50	4 MG	0.091	77.693	125.893	1991
		53	14 MG	0.322	79.223	122.885	3234
		54	3 MG	0.069	79.217	122.907	3054
		64	5MG; GKG	0.161	77.247	118.553	193
		68	7 MG	0.161	78.473	110.788	101
		69	6 MG	0.138	78.702	112.512	577
		70	14 MG; GKG	0.345	78.762	112.703	1216
		71	6 MG	0.138	78.582	111.387	235
ARK XXVII/3	Central	229	7 MG	0.161	83.997	31.318	4008
	Arctic	241	8 MG	0.184	83.932	76.711	3432
		262	6 MG	0.138	82.976	109.919	3601
		278	7 MG	0.161	82.883	129.955	4167
		326	8 MG	0.184	81.927	130.917	4038
		339	7 MG	0.161	85.057	122.736	4352
		355	8 MG	0.184	87.927	61.012	4381
		368	8 MG	0.184	88.787	56.372	4374
AMK-63	Laptev	5225	3 grabs	0.750	78.375	130.659	2390
	Sea	5227	3 grabs	0.750	78.091	130.521	2020

Subsamples: MG–multibox-corer; GKG–box-corer; grabs– 0.25 m^2^ Okean-grabs.

A total of eight macrobenthic stations were collected in the Nansen and Amundsen Basins during the RV *Polarstern* expedition ARK-XXVII/3 (August-September 2012). Sampling of macrofauna was conducted using the multibox-corer with six to eight 0.023 m^2^ subsamples taken at each station [31]. Subsamples were washed separately through a 0.5 mm mesh size sieve. Four cores were fixed with 4% formaldehyde and two to four cores (depending on the number of successfully closed cores) were fixed with 96% ethanol. Data on the biomass and density of macrobenthos based on this material were published by [6]. Our current study adds the taxonomic analysis. For additional information on sampling procedures, see [32].

Samples collected during the Cruise 63 of *Akademik Mstislav Keldysh* (September 2015) at the base of the Laptev Sea slope were also examined. Two stations using an *Okean* grab sampler with 0.25 m^2^ sampling area [33] were performed with three grab replicates per station. Each grab sample was washed through a 0.5 mm sieve and fixed by 5% formalin.

For all samples retrieved in 1993 and 2012, we assessed factors governing variations in macrobenthos composition including porosity, chloroplast pigments concentrations (chlorophyll *a* and phaeopigments), protein and phospholipids contents, bacterial abundance and activity (esterase and lipase activity) and organic carbon (S1 Table). For methods, see [34], and [35]. Samples for these parameters were obtained by multi-corer samples to retrieve the undisturbed top centimeter layer of sediment at the box core stations during the same expeditions. In addition we used the sea-ice coverage data measured at the moment of sampling at each station. Additional data from the Ocean Floor Observation System (OFOS) used in the ARK-XXVII/3 expedition allowed estimating the area of the *Melosira arctica* algal patches on the sediment surface (https://doi.pangaea.de/10.1594/PANGAEA.803293); for methods, see [9] and [31].

**Fig 1:**
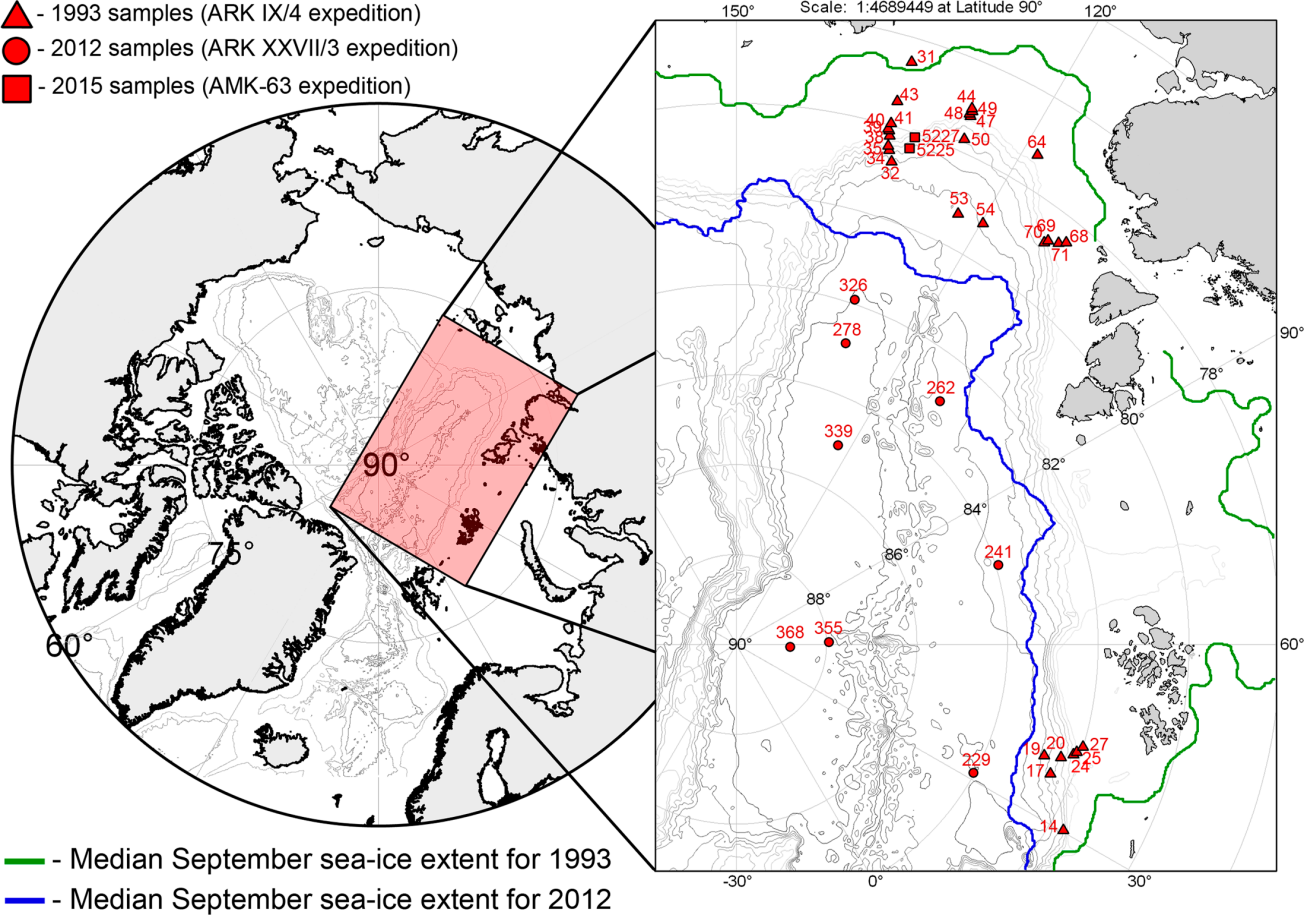
Sampling area, mean sea-ice extent and visited stations.

The map of the sampling area with stations is shown in Fig 1. Station data are given in Table 1. The environmental data used in this study are shown in S1 Table

Identification of macrofauna specimens to the lowest possible taxonomical level was made in the laboratory with the help of taxonomic experts (see ‘Acknowledgments’).

### Statistics

All specimens of each species were counted and weighted (wet weight, ww). We weighted all specimens of each species at a time. In case of molluscs the weight was measured together with shells. Polychaetes with mucous tubes (like *Spiochaetopterus typicus* or siboglinids) were weighted together with tubes. Sponges and ctenostomatid bryozoans were slightly blotted to remove extra water from their suface. Density and biomass were calculated per square meter. Dominant taxa in terms of abundance were distinguished for each station. The similarity between samples was estimated using the quantitative index of Bray-Curtis. We used the non-transformed data as a measure of taxonomic abundance because of the evenness across the most abundant taxa. Similarity matrixes were used for the cluster and multidimensional scaling (MDS) analyses. Clusters were generated using the UPGMA algorithm (unweighted pair group method). Results were verified by ANOSIM analysis to reveal different station groups. Species richness was estimated as a total number of species at each station. Taxonomic diversity was estimated using the Shannon-Wiener index and the Hurlbert rarefaction index for 100 individuals. The species-individuals accumulation curves for the fauna were plotted. Spearman’s rank correlation was calculated between the environmental variables and community/species characteristics. A canonical correspondence analysis (CCA) was performed to estimate the contribution of the sediment parameters, depth and ice-extent to taxonomic composition (i.e. species distribution within and between the samples) [36]. The differences between different years of sampling were tested by the similarity percentages routine (SIMPER). For consistency in depth zonation, only the stations taken at depths >2000 m were tested for decadal variation.

Statistical analyses were performed using the Microsoft Excel 2007 (calculating the biomass and abundance values), Primer V6 [37] (performed MDS, clusters, ANOSIM, diversity and SIMPER analyses) and PAST3 [38] (CCA and accumulation curves) software. Maps were generated using the PanMap software [39]

## Results

In total 10117 individuals (ind.) were obtained from 37 stations newly sorted for this study in a standardized manner. Most of the specimens were sampled in 1993 (9443 ind. in the 38–3234 m depth range; Table 1). New samples added 101 ind. from the 2012 campaign (3432–4381 m depth range) and 573 ind. from the campaign in 2015 (2020–2390 m depth range). The specimens were attributed to 440 taxa. The density of macrobenthos varied from 12 ind. m^-2^ (St. 229) to 24957 ind. m^-2^ (St. 31). The biomass ranged from 0.06 g ww m^-2^ (St. 229) to 650.53 g ww m^-2^ (St. 25). The list of identified taxa with density and biomass values at each station is given in S2 Table.

### Depth zonation of macrobenthos communities

**Fig 2:**
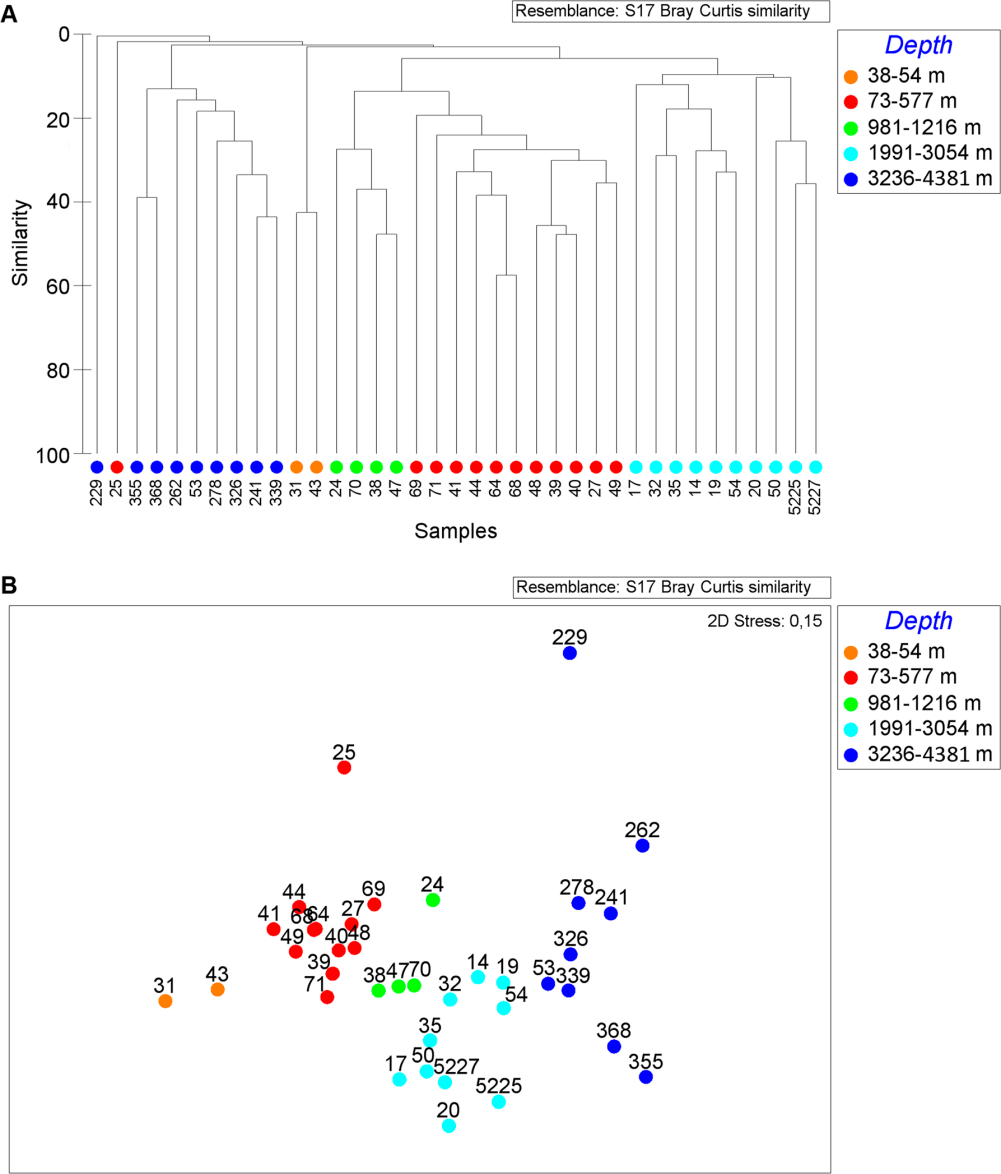
Cluster analysis (A) and non-metric multidimensional scaling plot (B) of all stations using the Bray-Curtis similarity index. Color indicates station group distinguished by depth. Orange–SHELF-group; red–UPPER SLOPE-group; green–MID-SLOPE-group; light-blue–LOWER SLOPE-group; dark-blue–PLAIN-group.

**Fig 3:**
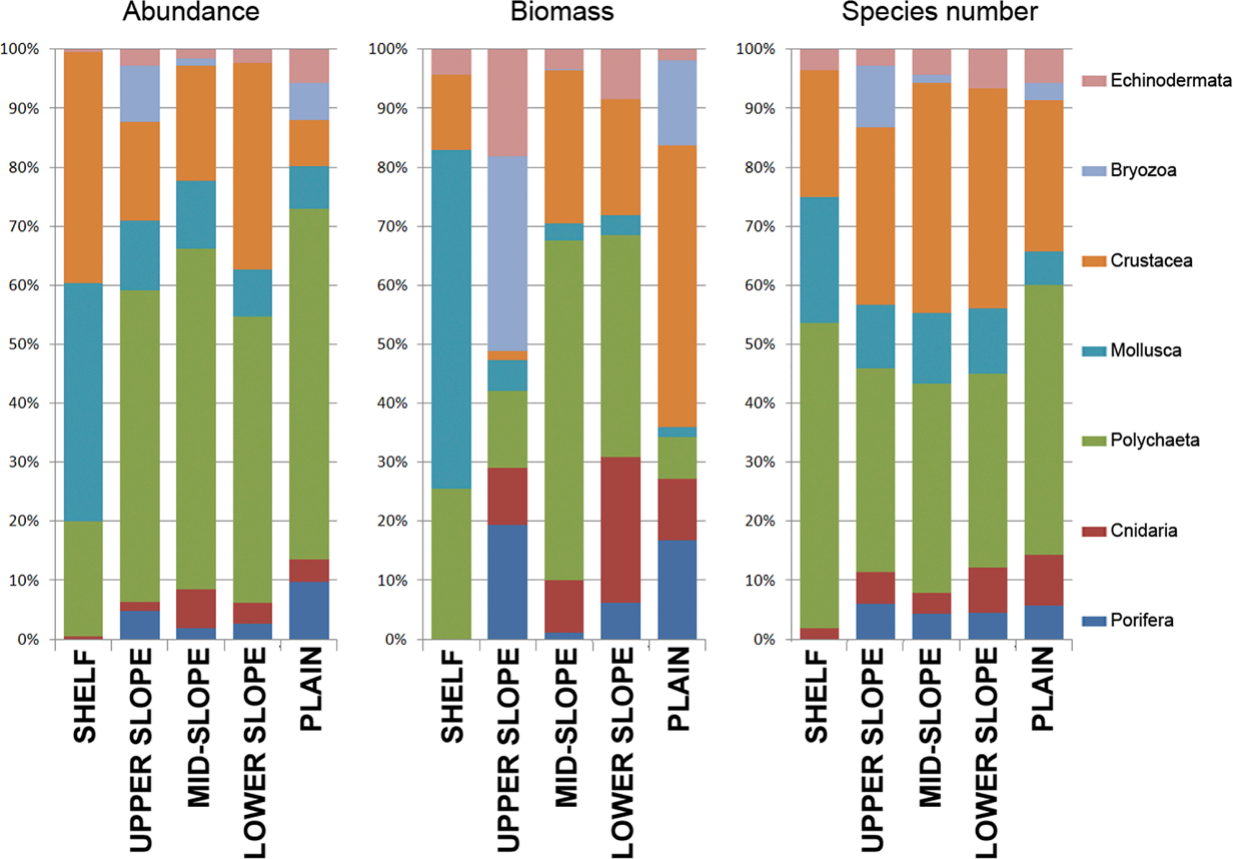
Percentage diagrams of total abundance, biomass and number of species per taxonomic group.

The non-metrical cluster analysis and multidimensional scaling revealed five distinct groups of macrobenthic communities distinguished by depth (Table 2, Fig 2). Between all zones, the maximum species overlap was 16% (Table 2). The community composition was significantly different (*p-*value of 0.001–0.022). The outer SHELF (38–54 m), UPPER SLOPE (73–1216 m), LOWER SLOPE (1991–3054) and PLAIN (3236–4381 m) groups were distinguished based on the Bray-Curtis similarity level (Fig 2A). We further distinguished as the fifth group the MID-SLOPE cluster (981–1216 m) from the UPPER SLOPE zone (73–577 m), because of the taxonomical peculiarity of the former (Fig 2, Table 3). The main dominants by density are shown in Table 3. Diversity characteristics of each group are given in Table 4. Species richness, contribution of each taxon to the total number of individuals, and to the total biomass within each station group are shown in Fig 3.

**Table 2:** Results of the ANOSIM analysis (abundance data). MID-SLOPE-group is included in the UPPER SLOPE-group.

Groups pairs	R statistic	Significance level (%)	Possible permutations	Actual permutations	% Shared taxa
SHELF–UPPER SLOPE	0.879	0.7	136	136	9.41
SHELF–LOWER SLOPE	1.000	1.5	66	66	3.68
SHELF–PLAIN	0.838	2.2	45	45	3.15
UPPER SLOPE–LOWER SLOPE	0.781	0.1	3268760	999	11.76
UPPER SLOPE–PLAIN	0.946	0.1	490314	999	4.55
LOWER SLOPE–PLAIN	0.700	0.1	43758	999	15.97

**Table 3:** Dominant taxa of the station groups. Depth range, number of stations in each station group, density of the dominant taxa and the mean dominance level with standard deviation are shown.

Station group	depth range (m)	number of stations	main dominant taxon	dominant taxon density (ind. m^-2^)	dominance (%)
SHELF	38–54	2	*Ennucula tenuis*	3859 ±1122	26.9 ±11.6
UPPER SLOPE	73–577	12	*Yoldiella solidula*	324 ±433	6.6 ±7.1
MID-SLOPE	981–1216	4	*Melinnopsis arctica*	165 ±68	14.9 ±6.5
LOWER SLOPE	1991–3054	10	*Galathowenia fragilis*	40 ±77	4.1 ±7.9
PLAIN	3236–4381	9	*Ymerana pteropoda*	10 ±13	10.7 ±13.4

**Table 4:** Diversity characteristics for different depth ranges. Mean values of species number, abundance, biomass, Pielou evenness, Hurlbert rarefaction index for 100 individuals and Shannon-Wiener index with standard deviation are shown.

Station group	Species per station	abundance (ind. m^-2^)	biomass (g ww m^-2^)	Pielou evenness	ES (100)	Shannon index
SHELF	35 ±23	16847 ± 11467	220.28 ±251.21	0.56 ±0.01	14.7 ±77.2	1.91 ±0.45
UPPER SLOPE	83 ±16	5293 ±2259	104.01 ±194.95	0.83 ±0.04	38.8 ±3.7	3.66 ±0.19
MID-SLOPE	64 ±32	1172 ±306	6.19 ±4.10	0.85 ±0.04	34.3 ±10.3	3.40 ±0.50
LOWER SLOPE	24 ±13	608 ±320	2.57 ±2.79	0.86 ±0.08	18.6 ±8.1	2.53 ±0.54
PLAIN	9 ±8	77 ±66	0.59 ±1.07	0.92 ±0.04	8.5 ±7.6	1.65 ±0.77

The SHELF group was characterized by the high dominance of bivalves *Portlandia arctica* and *Ennucula tenuis* and the ophiuroid *Ophiocten sericeum*. The biomass and density in this complex were the highest respectively, mostly owing to the bivalves (Fig 3, Table 4). This group contained only two stations. The SD-values of different community characteristics were the highest within this group (Tables 3 and 4).

The UPPPER SLOPE group encompassed 12 stations. Dominant species in terms of density and biomass in this group were the bivalve *Yoldiella solidula* and the polychaetes *Prionospio cirrifera* and Cirratulidae. Other notable species were the polychaetes *Proclea graffi* and *Notoroctus oculatus* and the cumacean *Ektonodiastylis nimia*. The biomass and density within this group were slightly lower than in the SHELF group, but diversity was the highest (Table 4). Polychaetes were the most abundant and diverse taxon, whereas the bryozoans and echinoderms (mainly the ophiuroids *Ophiacantha bidantata* and *Ophiocten sericeum*) contributed most to the biomass (Fig 3).

The MID-SLOPE group (four stations) was strongly dominated by the polychaete *Melinnopsis arctica* in terms of both the abundance and biomass. Other important species included the polychaete *Tharyx* sp., the bivalve *Yoldiella annenkovae*, the scyphozoan *Stephanoscyphus* and the sipunculid *Nephasoma diaphanes*. Crustacea was the most diverse taxon in this group (Fig 3). All diversity characteristics within the MID-SLOPE group, including the species number per station, density, biomass, ES (100) and Shannon index, were significantly lower than those in the UPPER SLOPE group (with the exception of Pielou evenness) (Table 4).

The LOWER SLOPE group included 10 stations. This group was rather uneven compared to others, and dominant species were different across stations, suggesting a substantial biodiversity variation at this water depth. The most abundant taxa included the polychaetes *Galathowenia fragilis*, *Ophelina opisthobranchiata* and *Terebellides* cf. *atlantis* and the tanaids *Pseudosphyrapus serratus* and *Pseudotanais* aff. *affinis*.

The PLAIN group (ten stations) was the least diverse among all station groups. The abundance, biomass, Shannon index and ES (100) were the lowest, whereas the Pielou evenness was the highest. Polychaeta was the most abundant and diverse taxon. Crustacea was more notable in terms of biomass, owing to the shrimp *Hymenodora glacialis* present in the catch at St. 53. This station near the Laptev Sea slope had the highest abundance, biomass and species number among the PLAIN group stations. The dominant taxa at stations of this group were different and included the polychaetes *Ymerana pteropoda* and *Anobothrus laubieri* and the sponge *Thenea abyssorum*. Other abundant taxon was the small bryozoan *Nolella* sp. Notable in samples of 2012 were scattered remains of semi-degraded ice-algae, apparently sedimented colonies of the diatom *Melosira arctica*.

Two stations, 25 and 229, were distinctly separate from other groups revealed by cluster analysis (Fig 2B). Station 25 of the northern Barents Sea slope within the depth range of the UPPER SLOPE group (534 m) differed from other stations by the prevalence of suspension-feeders, including Porifera, Hydrozoa, Serpulidae, Bryozoa and the ophiuroid *Ophiacantha bidantata* in terms of both density and biomass. The multi-box corer sample at St. 229 taken in the Nansen Basin at the depth of 4008 m (the PLAIN group depth range) contained only one colony of the bryozoan *Nolella* sp. and an ovum of an unknown invertebrate.

**Fig 4:**
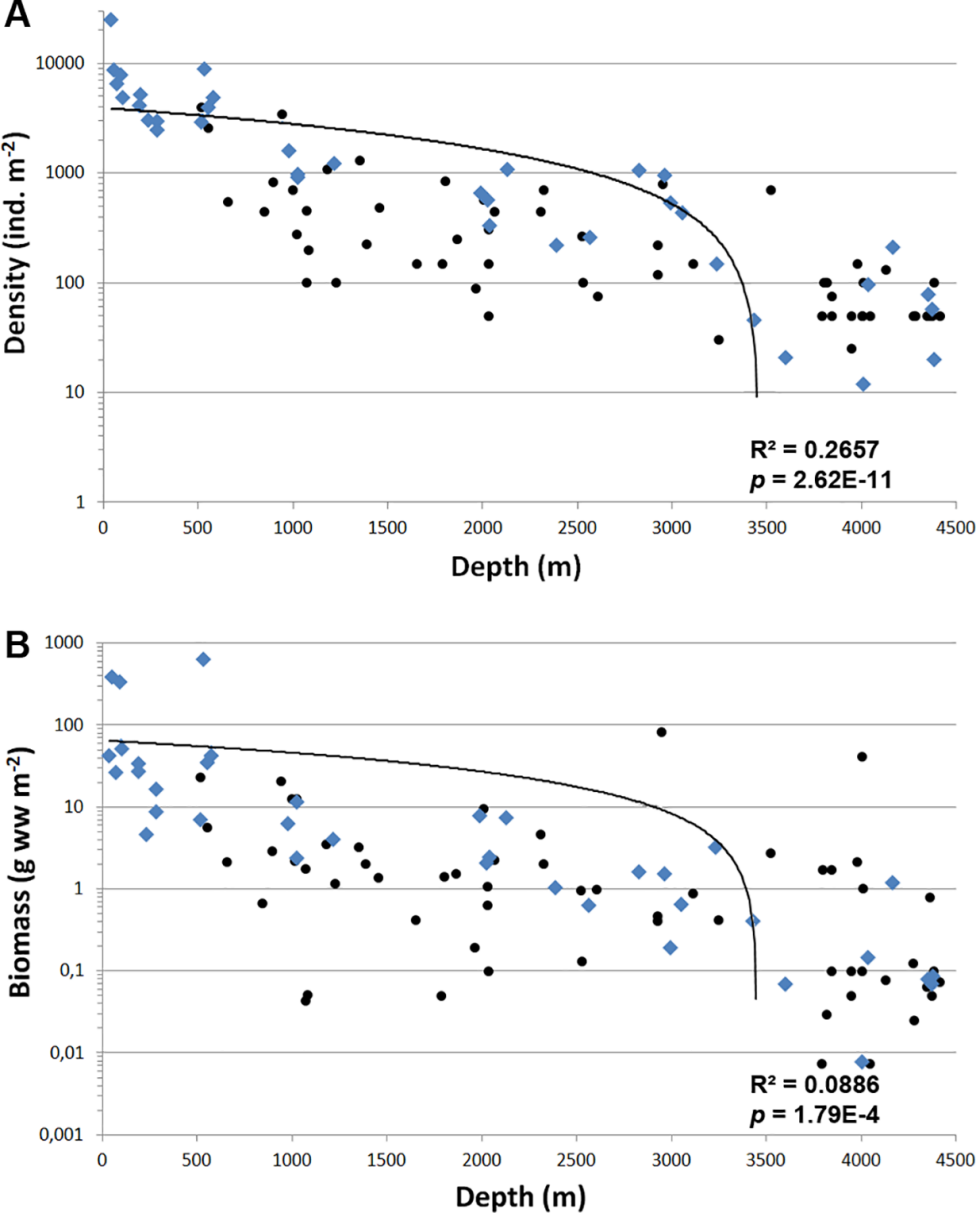
Abundance (A) and biomass (B) at stations in relation to depth. Blue rhombuses–our data; black dots–data from [6,28]. Lineal trend lines with R^2^ and *p* values calculated for all samples are added.

**Fig 5:**
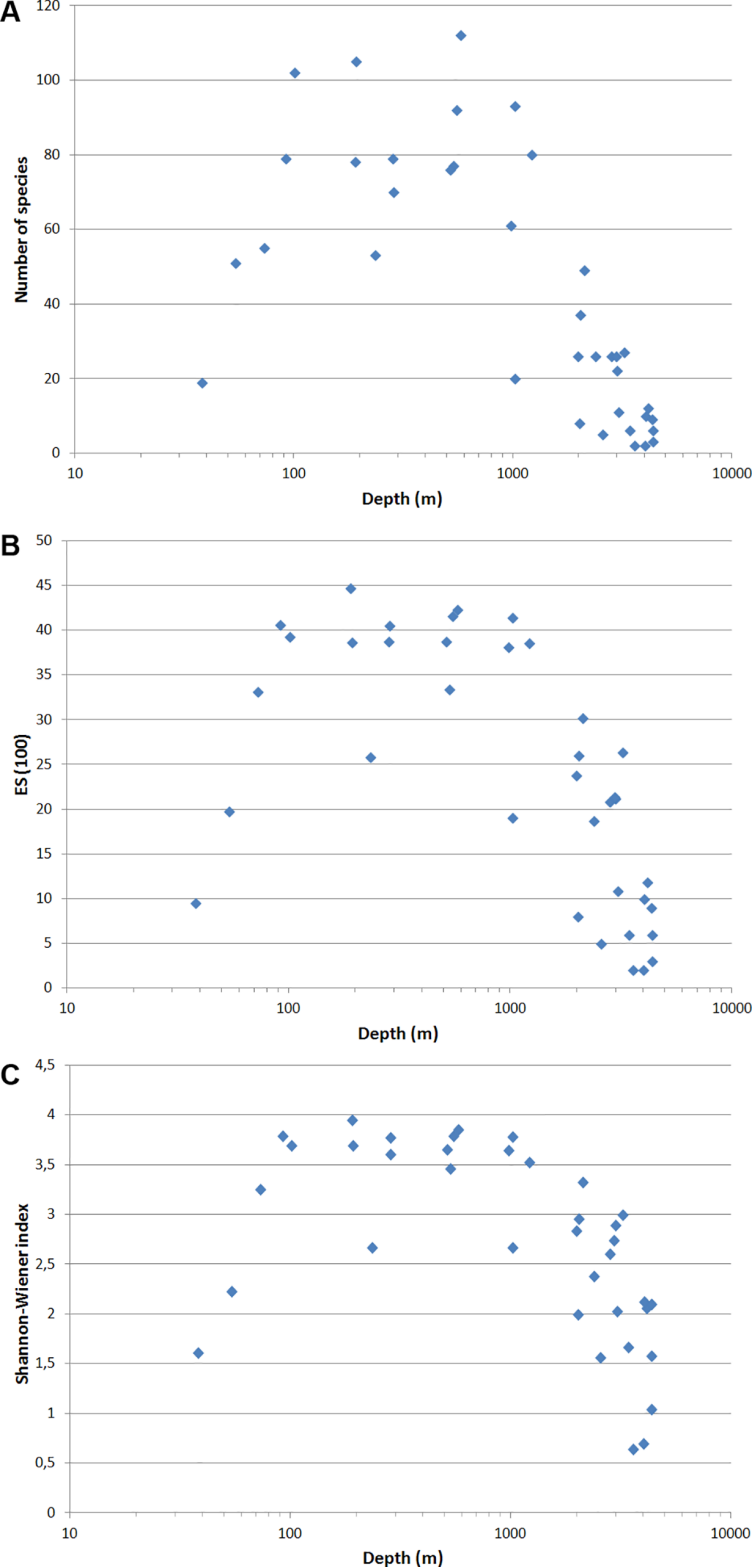
Number of species (A), Hurlbert rarefaction (B) and Shannon-Wiener index (C) at each station in relation to depth (logarithmic scale).

In general the biomass and density of macrobenthos distinctly decreased with depth from the shallow-most stations to the deepest (Fig 4A and 4B; Table 4). The results of the linear regression test for the abundance and biomass values are given in Table 5. The number of species, the Hurlbert rarefaction and the Shannon-Wiener index showed a maximum at the shelf break at depths ~100–300 m (Fig 5A, 5B and 5C).

**Table 5:** Results of the linear regression test for the abundance and biomass values related to depth. Data from 1991 [28], 1993, 1997 [6] and 2012–2015 are shown.

		Number of samples	R^2^	*p*-value
**Abundance**	all samples	96	0.2657	2.62E-11
	90-th samplings	86	0.2601	1.93E-10
	2000-th samplings	10	0.5315	0.0047
**Biomass**	all samples	96	0.0886	1.79E-04
	90-th samplings	86	0.0852	3.55E-04
	2000-th samplings	10	0.6079	0.0032

### Environmental factors governing macrobenthos distribution

**Fig 6:**
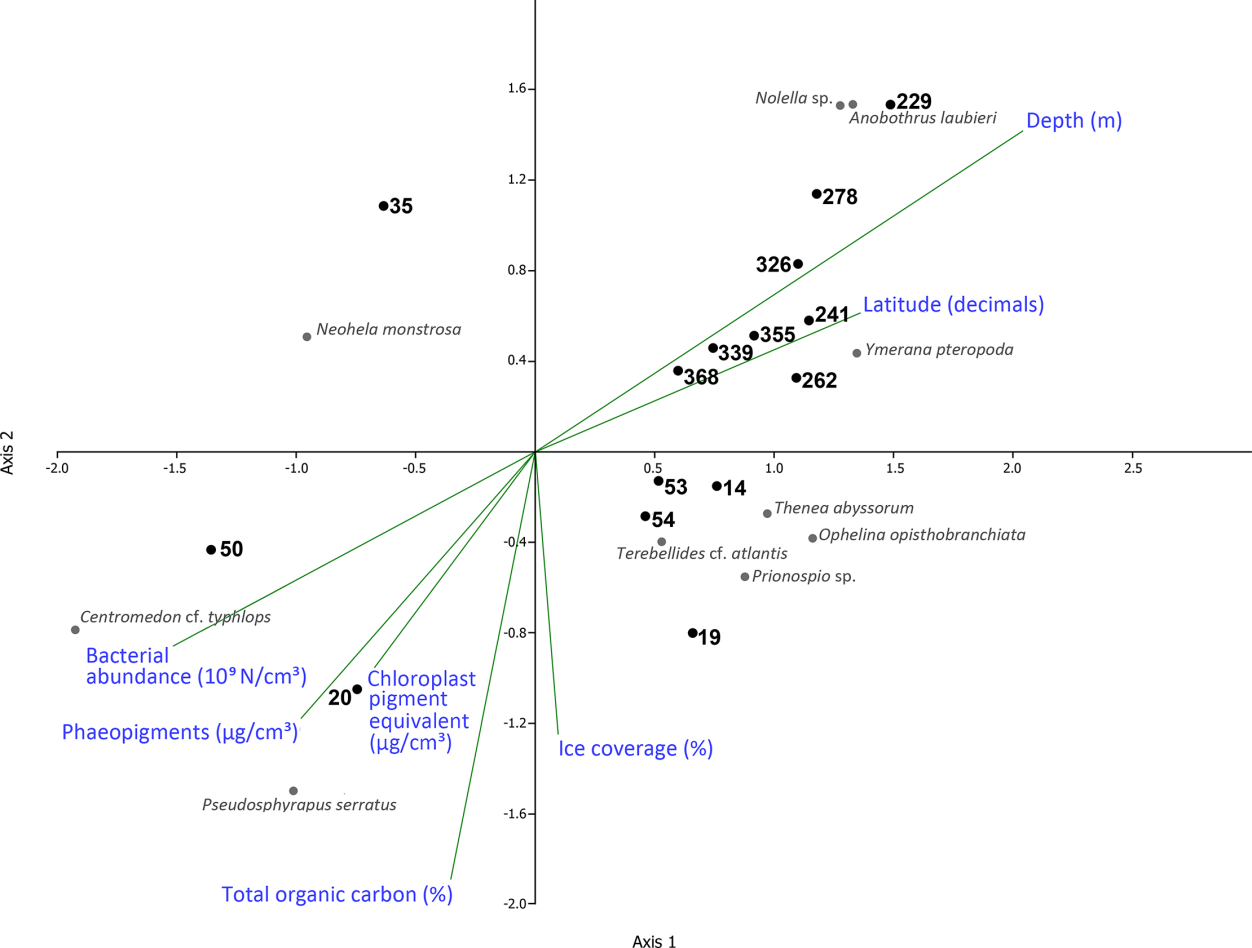
Results of canonical correspondence analysis (CCA) of the LOWER SLOPE and PLAIN station groups. Ten most abundant taxa are shown. Axis 1 significance level– 0.281; axis 2 significance level– 0.231.

Environmental data available for the sample collections of 1993 and 2012 allowed to test the correlation of total abundance of macrofauna with total chloroplastic pigments, indicating phytodetritus deposition, and with bacterial abundance in the surface sediments (Table 6). Of the individual taxa, the abundances of the tanaid *Pseudotanais affinis*, scaphopod *Siphonodentalium laubieri* and polychaete *Ophelina opisthobranchiata* correlated significantly in abundance with chloroplastic pigments, bacterial abundance and organic carbon concentration (Table 6). The results of canonical correspondence analysis among the deep stations (>2000 m, LOWER SLOPE and PLAIN groups) are shown in Fig 6. We chose the stations for which most of environmental variables were available for years 1993 and 2012. Stations 19, 20, 35 and 50 of the depth range 1991–2993 m demonstrated higher values of phaeopigments, bacterial abundance and organic carbon, and higher density of tanaid *Pseudosphyrapus serratus*, amphipod *Neohela monstrosa* and polychaete *Terebellides* cf. *atlantis*. The deep 2012 samples and the deepest 1993 station (53) showed low chloroplast pigments values, but higher densities of polychaetes *Ymerana pteropoda*, *Anobothrus laubieri* and bryozoan *Nolella* sp. (Fig 6). There was no direct correlation of these samples with the ice coverage, community composition and the abundance/biomass of distinct taxa.

**Table 6:** Spearman’s rank correlation between the community/species characteristics and the environmental/sediment parameters. Correlated pairs with p<0.05 are shown.

Correlated variables		Spearman's correlation	*p* (uncorr.)
Community characteristics/species	Environmental/ sediment parameter		
Abundance total	Depth	-0.80	0.0004
Abundance total	Php	0.78	0.0005
Abundance total	CPE	0.72	0.0026
Abundance total	Bact ab	0.88	1.84E-05
Biomass total	Depth	-0.74	0.0015
*Pseudotanais affinis* biomass	Depth	-0.81	0.0003
*Pseudotanais affinis* biomass	Php	0.78	0.0006
*Pseudotanais affinis* biomass	CPE	0.70	0.0036
*Pseudotanais affinis* biomass	Bact ab	0.81	0.0003
*Siphonodentalium laubieri* biomass	TOC	0.70	0.0037
*Ophelina opisthobranchiata* biomass	TOC	0.72	0.0025

For environmental/sediment parameters see S1 Table.

**Fig 7:**
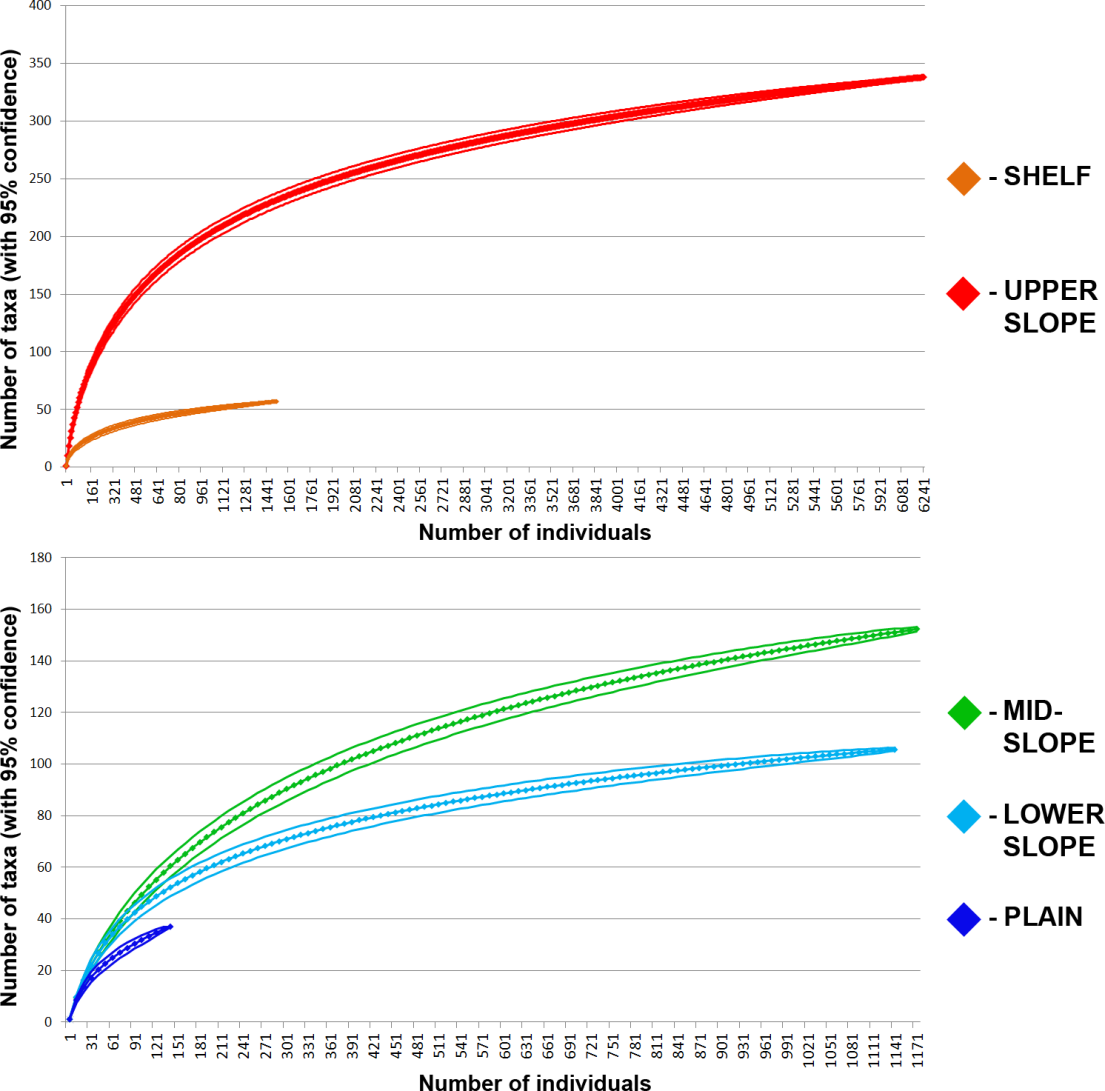
Species-individual accumulation curves with 95% confidence calculated separately for SHELF, UPPER SLOPE, MID-SLOPE, LOWER SLOPE and PLAIN station groups.

Species-individual accumulation curves for each station group are shown in Fig 7. The curves tend to reach the saturation in the SHELF and UPPER-LOWER SLOPE groups, whereas in the PLAIN group increasing the number of samples could significantly increase the total species numbers (Fig 7).

### Temporal trends

**Fig 8:**
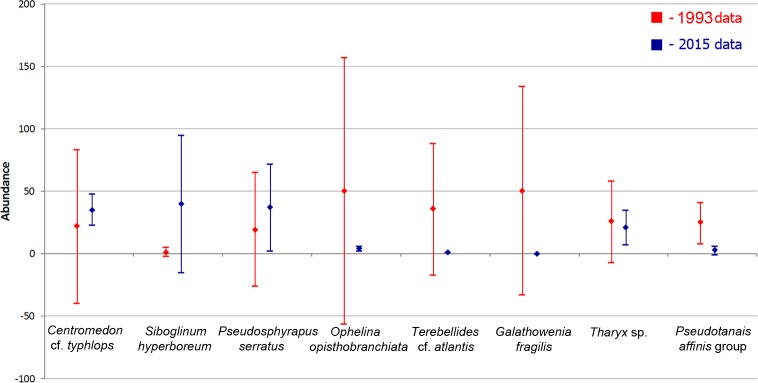
Abundance (mean values with standard deviation) of the species revealed by the SIMPER-analysis of 1993 and 2015 samples taken deeper than 2000 m.

To assess temporal trends, we selected 19 stations from all three expeditions with some regional overlap (depth >2000 m). The mean abundance of these taxa from 1993 and 2015 samples with the standard deviation are shown in Fig 8. Some differences in the abundance of certain taxa were revealed by SIMPER-analysis, but the standard deviation overlapped, so that no reliable conclusions about temporal changes could be made.

**Fig 9:**
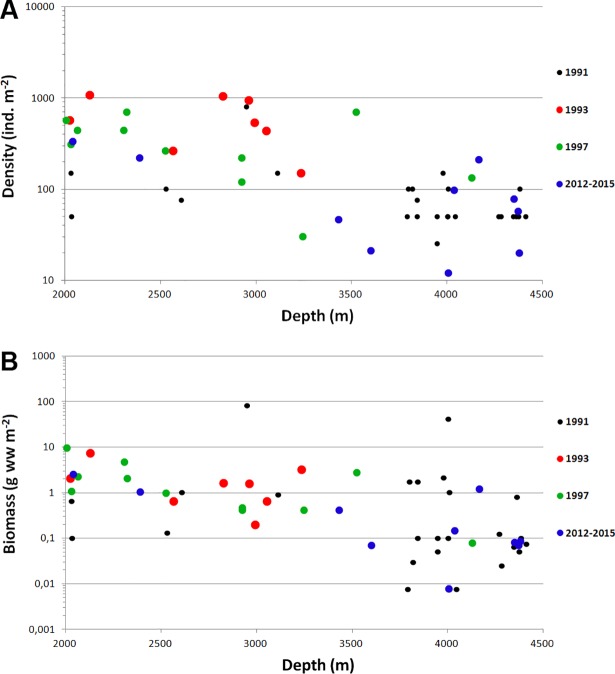
Abundance (A) and biomass (B) at stations in relation to depth (from 2000 to 4500 m). Data from 1991 [28], 1993, 1997 [6] and 2012–2015 are shown.

When limiting to total biomass and abundance and including the data of [5] and [6] for the depth zone 2000–4500 and comparing all data, no significant differences between the samples within these two decades (1991–1997 vs 2012–2015) could be detected (Fig 9).

## Discussion

### Macrobenthos of the Central Arctic Ocean is controlled by food availability

Similar to results of previous investigations that addressed factors influencing macrobenthos abundance and composition in other oceans, the present study revealed the substantial decrease of density and biomass between the seasonally ice-free shelves and the year round ice-covered basins [5,27,28]. We summarized published data on the biomass and abundance for the depth ranges used in our study (Table 7). It can be concluded that the ice-covered Central Arctic shows some of the lowest macrobenthos abundances and biomasses for the Northern Seas and the North Atlantic.

**Table 7:** Abundance and biomass of macrofauna at different geographical sites and different depth ranges. The values are arranged by latitude.

Region	Depth Ranges (m)						
	40–70 m	70–600 m	600–1300	1300–3000	3000–4200	Reference
	Abundance (ind./m^2^)					
This study	16847 ± 11467	5293 ±2259	1172 ±306	608 ±320	77 ±66	This study
Central Arctic	-	-	256 ±148	200 ±271	58 ±49	[13]
Central Arctic	-	2495 ±356	706 ±95	1042 ±854	349 ±341	[73]
Fram Strait, Central Arctic	-	4048	1294 ±1516	430 ±266	281 ±346	[6]*
Lomonosov Ridge	-	-	58 ±8	22 ±26	6 ±4	[11]
Canada Basin	-	-	50 ±26	78 ±65	-	[3]*
Canada Basin	-	4300	1867 ±383	1156 ±1061	113 ±62	[74]
Kara Sea slope	-	1988 ±1242	755	271 ±205	-	[16]
Laptev Sea slope	1220 ±732	2195 ±2924	420 ±150	516 ±434	252 ±133	[11]
Franz-Josef Land	6635 ±657	2785 ±1408	-	-	-	[41]
Off Spitsbergen, 79°N	-	2902 ±2180	-	646 ±240	-	[40]
Off Mauritania, 18–21° N	-	-	-	3984–5432	204–1840	[43]*
Goban Spur, 49° N	-	4785–7980	2882–6169	1413–1655	542–648	[44]
** **	**Biomass (g ww/m**^**2**^**)**					
This study	220.3 ±251.2	104.0 ±194.9	6.19 ±4.10	2.57 ±2.79	0.59 ±1.07	This study
Central Arctic	-	-	1.80 ±0.46	0.90 ±1.11	0.38 ±0.53	[13]
Central Arctic	-	-	0.55 ±0.39	0.40 ±0.19	0.14 ±0.20	[73]*
Fram Strait, Central Arctic	-	23,7	12.44 ±7.09	2.89 ±3.01	0.89 ±1.25	[6]*
Lomonosov Ridge	-	-	0.29 ±0.27	0.31 ±0.61	0.013 ±0.013	[11]
Canada Basin	-	-	0.11 ±0.07	0.64 ±1.08	-	[3]*
Kara Sea slope	-	643.8 ±995.8	28,1	2.35 ±0.05	-	[16]
Laptev Sea slope	82.3 ±51.2	33.4 ±34.2	3.68 ±2.97	1.75 ±1.35	0.34 ±0.47	[15]
Franz-Josef Land	3465 ±827.5	404.6 ±462.5	-	-	-	[39]
Off Spitsbergen, 79°N	-	38.7 ±14.5	-	2.20 ±1.50	-	[38]
Off Mauritania, 18–21° N	-	-	-	2.37 ±1.19	0.05 ±0.01	[40]
Goban Spur, 49° N	-	33.3–71.2	1.60–51.20	2.30–4.00	2.40–4.20	[41]*

*- the values include meiobenthos

In the shelf and slope areas similar values of abundance and biomass were reported for Laptev Sea [11,15], Fram Strait [40], Nansen Basin and Yermak Plateau [6]. Lower values were shown for the Lomonosov Ridge [11]. For the Arctic Ocean higher values of biomass were shown for the Kara Sea upper slope [16] and Franz-Josef Land [41] mostly due to sponges and other suspension-feeders. One of the reasons could be the courser sediment supporting higher biomass of encrusting sedentary organisms [42]. In the Atlantic Ocean much higher values of abundance were shown for the Mauritania slope [43] and for the Goban Spur area [44].

The global bathymetrical trends of biomass in the ocean were analyzed by [45]. The authors reported a biomass decrease from ~4 mg C m^-2^ on the Arctic shelf to <2.5 mg C m^-2^ in the Arctic deep sea, with a higher rate of decrease in the Arctic compared to the temperate and tropical areas of Atlantic and Pacific Oceans. The present study adds new data for the Central Arctic deep basins, confirming this trend of a more rapid decrease, most likely explained by low productivity and carbon export flux.

The biomass and density values observed at the abyssal stations (0.008–1.212 g ww m^-2^) agree with previously obtained data from other parts of the deep-sea Arctic [3,6,12]. [13,14] reported higher biomass and density values from the western parts of the Nansen and Amundsen Basins. However, this may result from samples contaminated by boreal shallow-water organisms, such as the polychaete *Magelona* sp., the amphipod *Jassa marmorata*, the bivalve *Spisula elliptica* etc. [13,14]. [15] suggested that these organisms could inhabit the pipeline system of a research vessel. After excluding those shallow-water species from the analysis, the biomass and density values appeared similar to those in our results (Table 7).

The overall decrease in abundance and biomass is connected with the food availability–the most important factor in determining the structure and function of benthic communities [46]. The amount of chloroplast pigments is commonly used as indicator of the particle flux and food availability in benthic ecosystems. It was shown that the amount of pigments decreases from the shelf edge towards abyssal depths in the Arctic Ocean [6,46]. In our study the abundance was strongly correlated with the phaeopigments and CPE values (Table 6).

Among 10 stations taken from >3200 m depth, the highest biomass and density were registered at the southern-most St. 53, the station the closest to the continental slope base. All other stations of this depth group were situated on the abyssal plain at some distance from the slope (>100 km from the 3000-isobath). Benthic biomass and abundance declined in the direction from the continental slope base towards poorer central abyssal areas. This effect in the Central Arctic can be explained by increasing sea-ice cover and limited primary productivity from restricted light availability [6,34,46].

During the *Polarstern* ARK27-3 cruise, numerous deposits of fresh diatom algae *Melosira arctica* were observed on the seafloor, including areas of all 2012 stations except for St. 229 [17]. This diatom was observed forming meter-long filaments on the under-ice surface in remarkable densities. Calculations showed that in the extreme year 2012 marked by the largest sea-ice melt recorded to date, algal deposition had reached an average 9 g C per m^2^ with *Melosira* contribution >85% of the carbon export. It was suggested that this significant increase in algal production could have resulted from recent climate changes affecting sea-ice and snow cover thickness, an important limiting factor for primary production [6,17,47].

These food falls of *Melosira* were shown to attract some of opportunistic megafaunal organisms, such as holothurians and ophiuroids [17]. Degraded parts of *Melosira* were discovered in some of 2012 samples. However, no evidence of direct macrobenthic response to algal biomass export to the seafloor was observed yet, and polychaete abundances were as low in 2012 as in previous years. However, the overall significantly positive correlation between the total abundance of macrobenthos and phaeopigments volume in our samples suggests that an overall increase in the sedimentation of phytodetritus is likely to increase faunal abundance and diversity in the future. Key indicator species for an increase in algal input could be tanaid *Pseudotanais affinis*; the biomass of this species is strongly correlated with phaeopigment concentrations (Table 6, Fig 8).

### Similarity of species composition is high across all depth zones

Previous studies of macrofauna community composition suggest a high proportion of endemic species in the Central Arctic with a large overlap in species between shallow and deep regions due to uniform temperature regime and other biogeographical factors [5,48]. Here we further investigated the hypothesis that despite large variations in abundance, a significant proportion of macrofaunal species within communities would be distributed independently of water depth in the Arctic. We used species richness and species diversity (the number of species per number of individuals) for comparison.

Species distribution in the Arctic is partially explained by the “polar emergence” of fauna (review in [49]). [49] distinguish two different phenomena termed in the literature the “polar emergence”: the extension of bathymetric ranges of recent bathyal and abyssal species in the Arctic to the shelf depths (i.e. the rise of deep-water species to shallow depths) (I) and the “evolutionary emergence and submergence” related to the colonization of different bathymetric zones in the history of shelf fauna (II). Both phenomena may increase the number of species at lower shelf–upper slope depth in the Arctic Ocean. Thus, [49] demonstrated that among 133 species occurring deeper than 2000 m in the Arctic, 27% can be found at depths between 200 and 1000 m and 41% at depths from 0 to 200 m (the most shallow depth of occurrence). This effect may also increase the number of species at upper slope–lower shelf depths. Our data show that 121 species found in LOWER SLOPE and PLAIN zones (e.g. deeper 2000 m, see doi.pangaea.de/10.1594/PANGAEA.890152) contribute 45% to the community composition (i.e. species representation) of MID-SLOPE zone, 47% to UPPER SLOPE and 5% to SHELF. However, beyond the similarity in presence/absence, the macrofauna composition showed a substantial dissimilarity of the different depth zones.

In the present study the macrobenthos clustered significantly according to water depth, despite pronounced differences in region, time, gears and washing protocols (Fig 2). Exceptions were one station of a high current regime at the shelf edge, and one almost devoid of fauna (Nansen Basin). The statistical test with ANOSIM supported that differences between the depth ranges were significant, and higher than the differences in species composition between particular samples from different areas (i.e. Barents and Laptev seas slope). The only other extensive study of benthic community composition in the Central Arctic also detected substantial water depth zonation [11]. This study was based on the samples of the RV *Polarstern* expedition ARK-XI/1 in 1995 and the IB *Oden* expedition Arctic Ocean-96 in 1996. Seven station groups were revealed by [11] on the eastern slope of the Laptev Sea, the Lomonosov Ridge and adjacent deep-sea parts of the Amundsen and Makarov Basins. The SHELF group distinguished in our study partly corresponds with the SHELF group shown by [11]. This group is characterized by benthic communities dominated by the bivalves *Ennucula tenuis* and *Portlandia arctica*, which are widely distributed at shallow depths of the Laptev and East-Siberian Seas [11,50,51]. However, since there are only two stations within this group, we refrain from making any conclusions about community characteristics. The diverse UPPER SLOPE group in our study agrees with the HANG (i.e. Slope) and RAND (i.e. Edge or Margin) station groups of [11]. This group is characterized by the *Ophiocten sericeum* and *Ophiopleura borealis* communities typical for the lower shelf–upper bathyal depths of the eastern Arctic [52–54]. In our study only two stations were collected on the outer shelf at depths <70 m (stations 31 and 43 forming the SHELF group) with a total sampled area 0.115 m^2^. The UPPER SLOPE group corresponding to the lower shelf–upper slope depths (73–577 m) consists of twelve stations with the total area sampled equivalent to 1.357 m^2^. It is obvious that increasing the number of stations from these depths would make conclusions more pronounced.

The MID-SLOPE group in our study matches the *Melinnopsis arctica* community described by [1] from the bathyal depths of the northern Kara Sea and the Lomonosov Ridge. [11] included stations from the similar depth range (981–1216 m) in the RAND group. The diverse LOWER SLOPE group in our study possibly corresponds to the RAND and RUCKEN (i.e. Ridge) communities described by [11].

The PLAIN group in our study matches the TIEF (i.e. Deep) group described by [11]. The species-individual accumulation curve for the PLAIN group suggests that the number of species will sharply increase with additional samples (Fig 7). At the same time, since the density of benthos in the deep-sea Central Arctic is lower than in other parts of the ocean [6,28], it can be assumed that the small sampling area of generally used quantitative gears (0.20–0.25 m^-2^) is not enough for the accurate estimation of regional species diversity.

Several taxa found in the PLAIN group have not been recorded in the Central deep-sea Arctic so far. The polychaete *Ymerana pteropoda* was previously known from one locality north from Svalbard [55], one locality in the Canada Basin north from Alaska [56] and two localities north-east off Iceland [57]. In our samples this species was present and even dominant in terms of density at five stations. In the genus *Nolella* (Bryozoa) the abyssal species were known only from the North Atlantic [58,59]. These peculiar bryozoans of a tentatively new species were present in two of our samples. These minute organisms could have been overlooked during previous investigations owing to their small size (1–3 mm) and dense silt coverage of zooids masking them among sediment particles [59].

We further investigated trends in macrobenthos diversity with water depth in comparison to other ocean areas. For the North Atlantic it was shown that the peak of species richness and diversity occurs at depths 2000–3000 m [26,60,61]. In the Goban Spur area of the north-east Atlantic species diversity increased with water depth from 208 m (ES(100) = 29) to 4115 m (ES(100) = 68) [25]. At the same site, the species richness per sample showed a different pattern, since the number of species is dependent on sample area, numerical density and species diversity [25]. [40] found that in Fram Strait, both species diversity and richness decreased with depth (depth range from 203 to 2977 m, ES(100) declined from 43 to 14). In the present study we detected a parabolic pattern of species diversity and richness change with depth, with the species maximum occurring at 100–300 m (ES(100) = 39 ±4), i.e. shallower than the bathymetric range investigated by [40] in Fram Strait.

A number of published studies on macrofauna on the Siberian shelf showed an increase of species richness and diversity from the upper shelf to the shelf edge [48,62–65]. Besides the explanation of this peak in diversity and richness by the “polar emergence” theory explained above, we also suggest that food availability by higher productivity of the marginal ice zone is a key factor. In the years 1980–2008 the seasonal ice zone was mostly found across the zones UPPER SLOPE and MID-SLOPE. Only recently it has retreated as far as beyond the BASE of the slope into the PLAIN [66]. It remains an important task to continue surveys of potential benthic community shifts in response to the retreating sea-ice extent in the Eurasian sector of the Arctic Ocean.

The comparably lower macrobenthos richness and biomass on the Siberian Arctic shelf could be a regional phenomenon, related to the massive river run-off, potentially impacting ocean stratification and thereby food availability. The Ob, Yenisei and Lena rivers contribute almost 1700 km^3^ of fresh water per year into the Kara and Laptev Seas, which is about 60% of the total Arctic rivers annual discharge [67,68]. For the Kara Sea it was shown that brackish waters generated by the rivers Ob and Yenisei add to the stratification of the shelf sea and form a thick and extensive layer on the surface. The layer spreads in the form of giant lenses and can be traced even along the east coast of the Novaya Zemlya Islands [69]. The layer of brackish waters reduces nutrient transport in summer [70]. This may explain the lower species richness of benthos in the middle of the shelf compared to the shelf edge. In the Laptev Sea a peak in primary production related clearly to the border of the freshwater lens was found near the shelf-edge above the depths 100–200 m [71]. Another possible reason of the lower diversity on the shallower parts of Siberian shelf could be the sea-ice scraping. Large furrows on the sediment surface left by sea ice can be traced down to 15–20 m depth [72]. However, the influence of ice scraping on benthic macrofauna has not been studied in our work.

### Temporal changes

In the present study we synthesized and tested temporal and regional differences in benthic data in a Panarctic perspective ([5,28], this study). However, it remains currently impossible to assess potential temporal trends of the macrobenthos in response to the rapid sea-ice decline of this decade, due to the low number of benthic samples available from the Arctic Ocean (Fig 8). If temporal variation did exist, spatial variation would completely mask it. Comparison of the total abundance and biomass data revealed no differences between different years of samplings (Fig 9). However, the set of stations collected in the Central Arctic deeper than 2000 m is restricted. Repeated surveys from the same areas are needed for the correct analysis of the potential decadal changes in macrobenthos community composition in the Nansen and Amundsen basins. Furthermore, a taxonomical revision and standardizing of previously published species lists [11,15,16,73] by one pool of taxonomical experts is required.

## Conclusions

The present work has demonstrated that on the slope of the Barents and Laptev Seas and the abyssal of the Nansen and Amundsen Basins, the macrobenthos communities group according to water depth. Five station groups were distinguished based on species composition analysis in the depth range from 38 m to 4381 m: 1) shelf (38–54 m); 2) lower shelf and upper slope (73–577 m); 3) mid-slope (981–1216 m); 4) lower slope (1991–3054 m); and 5) abyssal plain (3236–4381 m). These groups partly correspond to the groups described by [11] and are apparently widely distributed along the Arctic slope and abyssal plains. The distribution of the groups is strongly correlated with depth and group boundaries do not overlap bathymetrically. We partly confirmed that some variation in macrobenthos distribution is controlled by food availability. According to our data, the total macrofaunal density is correlated with the amount of phaeopigments.

The polychaete *Ymerana pteropoda* and the bryozoan *Nolella* sp. were recorded in open areas of Nansen and Amundsen Basins for the first time. Rarefaction analyses predict that the existing diversity of slope and basin macrobenthos has not been adequately sampled yet.

In this study, 37 samples collected in three different expeditions using three different sampling and washing techniques were analyzed. Despite these differences, the samples appeared to be comparable. However, more uniform sampling techniques should be used for the correct analysis including larger sampling area at each station. Identification of macrofaunal organisms should be performed by the same specialists in order to exclude misidentifications.

Decrease of density and biomass of macrobenthos with depth was confirmed for the Central Arctic Basin. The depth-related parabolic pattern of species diversity with the maximum at 100–300 m was shown for the Arctic for the first time. Temporal changes caused by thinning and retreating sea ice, which could potentially enhance primary productivity and export fluxes, are currently impossible to ground-truth given the lack of data for the Arctic Ocean deep seafloor.

## Supporting information

S1 Table Sediment characteristics and ice coverage at the stations https://doi.pangaea.de/10.1594/PANGAEA.890151. (DOC)

S2 Table Macrofaunal abundance and biomass. https://doi.pangaea.de/10.1594/PANGAEA.890149 https://doi.pangaea.de/10.1594/PANGAEA.890150.
(XLS)
