# Supplementary material for: Spatial distribution of benthic macrofauna in the Central Arctic Ocean
Source: PLoS One. 2018 Oct 17;13(10):e0200121. doi: 10.1371/journal.pone.0200121 (PMC6192560; doi:10.1371/journal.pone.0200121)
Supplement: S1 Table — (DOC) [file pone.0200121.s001.doc]

S1 Table. Sediment characteristics and ice coverage at the stations

| **Station** | **Por** | **Prot** | **Est** | **Lip** | **Phosph** | **Chl *a*** | **Php** | **CPE** | **TOC** | **Bact ab** | **Alg** | **Ice** |
| --- | --- | --- | --- | --- | --- | --- | --- | --- | --- | --- | --- | --- |
| 14 | 0.5 | 133.57 | 3460.1 | 0.046 | 15.17 | 0.409 | 2.29 | 2.699 | 1.85 | 2.28 | - | 95.0 |
| 17 | - | - | - | - | - | - | - | - | 1.81 | - | - | 95.0 |
| 19 | 0.9 | 106.61 | 1933.6 | 0.084 | 5.35 | 0.038 | 1.17 | 1.208 | 1.69 | 1.28 | - | 95.0 |
| 20 | 0.8 | 134.79 | 4002.7 | 0.031 | 7.01 | 0.053 | 1.59 | 1.643 | 1.63 | 1.81 | - | 95.0 |
| 24 | - | 126.90 | 4589.5 | - | 9.27 | 0.054 | 1.67 | 1.724 | 1.54 | 1.66 | - | 95.0 |
| 25 | - | 125.51 | - | 0.026 | - | 0.080 | 2.44 | 2.520 | 2.01 | 0.13 | - | 95.0 |
| 27 | 0.9 | 122.72 | 5902.9 | 0.173 | 10.65 | 0.197 | 2.67 | 2.867 | 0.40 | 0.87 | - | 95.0 |
| 31 | 0.8 | 395.18 | 4785.7 | 0.054 | 16.51 | 4.556 | 4.79 | 9.346 | 1.20 | 1.47 | - | 0.0 |
| 32 | 0.8 | 113.93 | 3460.1 | 0.000 | 7.38 | 0.044 | 0.87 | 0.914 | - | 2.05 | - | 0.0 |
| 35 | 0.8 | 74.16 | 1864.3 | 0.045 | 9.20 | 0.048 | 1.14 | 1.188 | 0.33 | 1.85 | - | 0.0 |
| 38 | 0.9 | 133.49 | 2369.7 | 0.069 | 11.97 | 0.069 | 2.05 | 2.119 | 1.19 | 1.11 | - | 5.0 |
| 39 | - | 204.13 | - | 0.063 | - | 0.152 | 2.21 | 2.362 | 1.18 | 2.29 | - | 0.0 |
| 40 | - | 300.19 | 4629.7 | 0.038 | - | 0.227 | 2.51 | 2.737 | 0.81 | 2.13 | - | 0.0 |
| 41 | - | - | - | - | - | - | - | - | 0.87 | - | - | 5.0 |
| 43 | - | 586.47 | 15533.2 | 0.233 | - | 1.762 | 11.38 | 13.142 | 1.31 | 3.62 | - | 40.0 |
| 44 | 0.6 | 403.37 | 1394.1 | 0.060 | 17.03 | 0.197 | 1.87 | 2.067 | 0.34 | 1.63 | - | 60.0 |
| 47 | 0.9 | 191.98 | 1085.2 | 0.054 | 12.92 | 0.034 | 1.71 | 1.744 | 1.40 | 0.95 | - | 90.0 |
| 48 | 0.8 | 314.93 | 1816.5 | 0.124 | 11.91 | 0.108 | 2.15 | 2.258 | 1.00 | 0.56 | - | 75.0 |
| 49 | 0.6 | 301.99 | 2146.8 | 0.000 | 16.06 | 0.227 | 2.16 | 2.387 | 0.29 | 1.85 | - | 90.0 |
| 50 | 0.9 | 314.93 | 3456.2 | 0.014 | 8.26 | 0.048 | 1.29 | 1.338 | 1.10 | 1.98 | - | 90.0 |
| 53 | 0.9 | 86.28 | 7168.2 | 0.037 | 8.10 | 0.017 | 0.54 | 0.557 | 1.22 | 0.35 | - | 95.0 |
| 54 | - | 104.36 | 5271.8 | 0.180 | 7.70 | 0.005 | 0.83 | 0.835 | 1.52 | 1.34 | - | 75.0 |
| 64 | 0.71 | 378.41 | 2635.7 | 0.077 | 20.98 | 0.182 | 2.65 | 2.832 | 0.57 | 1.89 | - | 80.0 |
| 68 | - | 298.39 | - | 0.284 | - | 0.278 | 3.39 | 3.668 | 0.56 | 4.62 | - | 95.0 |
| 69 | - | - | - | - | - | - | - | - | - | - | - | 100.0 |
| 70 | - | - | - | - | - | - | - | - | 0.45 | - | - | 100.0 |
| 71 | - | - | - | - | - | - | - | - | 0.66 | - | - | 100.0 |
| 229 | 0.74 | - | - | - | - | 0.072 | 0.62 | 0.693 | 0.78 | 0.84 | 0.00 | 81.5 |
| 241 | 0.79 | - | - | - | - | 0.244 | 1.19 | 1.434 | 0.97 | 1.04 | 0.03 | 56.4 |
| 262 | 0.79 | - | - | - | - | 0.212 | 0.76 | 0.972 | 0.91 | 0.95 | 1.59 | 100.0 |
| 278 | 0.86 | - | - | - | - | 0.220 | 0.80 | 1.020 | 0.77 | 1.15 | 0.30 | 78.7 |
| 326 | 0.91 | - | - | - | - | 0.125 | 0.58 | 0.706 | 0.94 | 1.17 | 0.48 | 4.0 |
| 339 | 0.82 | - | - | - | - | 0.070 | 0.42 | 0.490 | 0.77 | 0.88 | 0.40 | 62.3 |
| 355 | 0.71 | - | - | - | - | 0.080 | 0.50 | 0.580 | 0.36 | 0.78 | 2.37 | 85.8 |
| 368 | 0.81 | - | - | - | - | 0.120 | 0.69 | 0.810 | 0.55 | 0.87 | 10.31 | 100.0 |
| 5225 | - | - | - | - | - | - | - | - | - | - | - | 0 |
| 5227 | - | - | - | - | - | - | - | - | - | - | - | 0 |

Por – Sediment water contents (%); Prot – protein volume (µg/ml); Est – esterase activity per sediment volume (nmol/ml/h); Lip – lipase activity per sediment volume (nmol/ml/h); Phosph – phospholipids per sediment volume (nmol/ml); Chl *a* – chlorophyll *a* per sediment volume (µg/cm^3^); Php – phaeopigments per sediment volume (µg/cm^3^); CPE – chlorophyll pigment equivalent (µg/cm^3^); TOC – total organic carbon in sediment (%); Bact ab – bacterial abundance as single cells per sediment volume (10^9^ N/cm^3^); Alg – *Melosira arctica* algae coverage of the sediment surface (%); Ice – ice coverage (%); hyphen – no data.
